# On bounds in Poisson approximation for distributions of independent negative-binomial distributed random variables

**DOI:** 10.1186/s40064-016-1710-y

**Published:** 2016-01-26

**Authors:** Tran Loc Hung, Le Truong Giang

**Affiliations:** University of Finance and Marketing, 306 Nguyen Trong Tuyen Street, Tan Binh District, Ho Chi Minh City, Vietnam

**Keywords:** Stein–Chen method, Poisson approximation, Le Cam’s inequality, Negative-binomial variable, 60F05, 60G50, 41A36

## Abstract

Using the Stein–Chen method some upper bounds in Poisson approximation for distributions of row-wise triangular arrays of independent negative-binomial distributed random variables are established in this note.

## Background

Let $X_{n, 1}, X_{n, 2}, \ldots ; n=1, 2, \ldots$ be a row-wise triangular array of independent negative-binomial distributed random variables with probabilities

1 $$P\left( X_{n, i} = k \right) = C_{r_{n, i} + k - 1}^{k} \left( 1-p_{n, i} \right) ^{k}p_{n, i}^{r_{n,i}},$$

where $p_{n, i}\in (0,1); r_{n,i}=1, 2, \ldots ; i=1, 2, \ldots ; k=0, 1, \ldots .$ It is worth pointing out that if all $r_{n,1}=r_{n, 2}=\cdots =1; n=1, 2, \ldots ,$ then we have the sequence of independent geometric distributed random variables with success probabilities $p_{n, 1}, p_{n, 2}, \ldots ; n=1, 2, \ldots .$ Write $W_{n}=\sum \nolimits _{i = 1}^{n} X_{n, i}$ and ${\lambda _n} =E\left( W_{n} \right) =\sum \nolimits _{i = 1}^{n} r_{n,i} \left( 1-p_{n, i} \right) p_{n, i}^{-1}.$ We will denote by $Z_{\lambda _{n}}$ the Poisson random variable with positive mean $\lambda _{n}.$

The main aim of this paper is to establish some upper bounds in Poisson approximation for $\sum \nolimits _{k=1}^{\infty }\mid P(W_{n}=k)-P(Z_{{\lambda _n}}=k) \mid$ for the sequence $X_{n, 1}, X_{n, 2}, \ldots ; n=1, 2, \ldots$ by the well-known Stein–Chen method.

It has long been known that the remarkable Le Cam’s inequality in Poisson approximation for the row-wise triangular array of independent Bernoulli distributed random variables $Y_{n, 1}, Y_{n, 2}, \ldots ; n=1, 2, \ldots$ with probabilities $P(Y_{n, i}=1)=p_{n, i}=1-P(Y_{n, i}=0), i=1, 2, \ldots$ is defined as follows:

2 $$\sum_{k=1}^{\infty } \mid P(S_{n}=k)-P(Z_{\beta _{n}}=k) \mid\,\le 2 \sum _{i=1}^{n}p_{n, i}^{2},$$

where $S_{n}=\sum _{i=1}^{n}Y_{n, i}$ and $\beta _{n}=E(S_{n})=\sum \nolimits _{i=1}^{n}p_{n, i}$ [see Le Cam (1960), Neammanee (2003) for more details]. Moreover, a shape inequality has been established as follows:

3 $$\begin{aligned} \sum \limits _{k=1}^{\infty } \mid P(S_{n}=k)-P(Z_{\beta _{n}}=k) \mid\, \le \frac{2(1-e^{-\beta _{n}})}{\beta _{n}}\sum \limits _{i=1}^{n}p_{n, i}^{2}. \end{aligned}$$

[We refer the reader to Barbour et al. (1992) and Chen (1975)]. As far as we know the Stein–Chen method is the well-known method have been used in Poisson approximation problems and it can be applied to a wide class of discrete random variables as geometric distributed random variables and negative-binomial distributed random variables. In recent years, using the Stein–Chen method, many results related to Poisson approximation for various discrete random variables are established in Teerapabolarn and Wongkasem (2007), Teerapabolarn (2009, 2013). These results are included here for the sake of completeness. Let $Z_{1}, Z_{2}, \ldots$ be a sequence of independent geometric distributed random variables with probabilities $P(Z_{i}=k)=(1-p_{i})^{k}p_{i}, k=0, 1, 2, \ldots ; i=1, 2, \ldots$ Then, for $A\subseteq \mathbb {Z}_{+}:=\{0, 1, 2, \ldots \},$

4 $$\mathop {\sup }\limits _A\mid P(V_{n}\in A)-\sum _{k\in A}\frac{{\gamma _n}^{k}e^{-{\gamma _n}}}{k!}\mid \,\le \sum _{i=1}^{n}\min \left\{ \frac{{\gamma _n}^{-1}(1-e^{-{\gamma _n}})}{p_{i}}, 1 \right\} (1-p_{i})^{2}p_{i}^{-1},$$

and for $$A\subseteq \mathbb {Z}_{+}, w_{0}\in \mathbb {Z}_{+}$$

5 $$\mid P(V_{n}\le w_{0})-\sum _{k= 0}^{w_{0}}\frac{{\gamma _n}^{k}e^{-{\gamma _n}}}{k!}\mid \le {\gamma _n}^{-1}(e^{-{\gamma _n}}-1)\sum _{i=1}^{n}\min \left\{ \frac{1}{p_{i}(w_{0}+1)}, 1 \right\} (1-p_{i})^{2}p_{i}^{-1},$$

where $V_{n}=\sum _{i=1}^{n}Z_{i}, \gamma _{n}=E(V_{n})=\sum _{i=1}^{n}(1-p_{i})p_{i}^{-1}$ [see Teerapabolarn and Wongkasem (2007), for more details]. It should be noted that in case when the mean ${\gamma _n}=E(V_{n})$ will be replaced by a parameter $\bar{{\gamma _n}}=\sum _{i=1}^{n}(1-p_{i}),$ another results will be established as follows:

6 $$- \bar{{\gamma _n}}^{-1}(e^{-\bar{{\gamma _n}}}-1)\sum _{i=1}^{n}\min \left\{ \frac{1}{p_{i}(w_{0}+1)}, 1 \right\} (1-p_{i})^{2} \le P(V_{n}\le w_{0})-\sum _{k=0}^{w_{0}}\frac{\bar{{\gamma _n}}^{k}e^{-\bar{{\gamma _n}}}}{k!}\le 0,$$

and

7 $$\begin{aligned}&\mid P(V_{n}\le w_{0})-\sum _{k=0}^{w_{0}} \frac{e^{-\bar{{\gamma _n}}}\bar{{\gamma _n}}^{k}}{k!}\mid \nonumber \\&\quad \le \frac{\sum _{k=0}^{w_{0}}\frac{e^{-\bar{{\gamma _n}}}\bar{{\gamma _n}}^{k}}{k!}(1-\sum _{k=0}^{w_{0}} \frac{e^{-\bar{{\gamma _n}}}\bar{{\gamma _n}}^{k}}{k!})}{\frac{{{e^{\bar{{\gamma _n}}}}{\bar{{\gamma _n}}}^{{w_0} + 1}}}{{\left( {{w_0} + 1} \right) !}}} \sum _{i=1}^{n}\min \left\{ \frac{1}{p_{i}(w_{0}+1)}, 1 \right\} (1-p_{i})^{2}, \end{aligned}$$

for $A\subseteq \mathbb {Z}_{+}$ [results of this nature may be found in Teerapabolarn (2013)]. It is easy to check that when the values $r_{n,1}=r_{n, 2}=\cdots =1; n=1, 2, \ldots$ the desired sequence $(X_{n}, n\ge 1)$ will become the sequence $Z_{1}, Z_{2}, \ldots .$ Therefore, it makes sense to consider the results in (4), (5), (6), and (7) for negative-binomial random variables with probabilities in term of (1).

It should be noted that in recent years the same problem was tackled in Upadhye and Vellaisamy (2014) and Vellaisamy and Upadhye (2009) by using Kerstans method (1964) and the method of exponents [see Upadhye and Vellaisamy (2013, 2014) and Vellaisamy and Upadhye (2009), for more details]. The compound negative binomial and compound Poisson approximations to the generalized Poisson binomial distribution are studied and applications are also discussed [see Upadhye and Vellaisamy (2013, 2014), for more details]. Specifically, using Kerstans method (1964) and the method of exponents, Vellaisamy and Upadhye (2009) have established the bounds in Poisson approximation as following inequality:

$$d_{TV}\left( S_{n}, Z_{\lambda } \right) \le \sum \limits _{j=1}^{n}\frac{\alpha _{j}q^{2}_{j}}{p_{j}}\min \left \{1, \frac{1}{\sqrt{2\lambda e}} \right \},$$

where $\lambda =\sum \nolimits _{i=1}^{n}\alpha _{i}q_{i}=\alpha q,$ for $X_{1}, X_{2}, \ldots , X_{n}$ are independent negative binomial distributed random variables with parameters $\alpha _{j}$ and $q_{j}, j=1, 2, \ldots , n$ and $Z_{\lambda }$ is a Poisson random variable with mean $\lambda .$

It is worth pointing out that comparison of bounds in negative binomial approximation and Poisson approximation is showing that an negative binomial approximation is better than Poisson approximation in the case $X_{j}, j=1, 2, \ldots$ are independent negative binomial random variables [see Theorem 2.2 and Theorem 2.4 in Vellaisamy and Upadhye (2009)].

Besides, Poisson approximation is also considered for a wide class of discrete random variables via operator method and method of probability distance [see Hung and Thao (2013) and Hung and Giang (2014), for more details].

The main purpose of this paper is to use the Stein–Chen method for providing the bounds of Le Cam-type inequality (2) and (3) in Poisson approximation for row-wise arrays of independent negative-binomial distributed random variables. The results obtained in this paper are extensions and generalizations of some results in Teerapabolarn and Wongkasem (2007), Teerapabolarn (2009, 2013).

## Preliminaries

During the last several decades the Stein–Chen method has risen to become one of the most important tools available for studying in Poisson approximation problems. The Stein–Chen method has been dealt with in detail in many articles [the reader is referred to Stein (1972), Chen (1975), Chen and Röllin (2013), Barbour et al. (1992) and Barbour and Chen (2004) for fuller development]. The Stein–Chen method can be summarized as follows:

Let us denote by $F_{X}(A)$ the probability distribution function of a discrete random variable $X\in A$ and we will denoted by $P_{\alpha _n} \left( A \right) = \displaystyle \sum \nolimits _{k \in A} {{e^{ - {\alpha _n} }}\frac{{{{\alpha _n} ^k}}}{{k!}}}$ the Poisson distribution function, defined on the set $A\subseteq \mathbb {Z}_{+}.$ The best known method for estimating

$$\begin{aligned} \Delta = \mathop {\sup }\limits _x \left| {F_{X}\left( A \right) - P_{\alpha _n} \left( A \right) } \right| \end{aligned}$$

is basing on the following arguments [see Chen (1975) for more details]:

Assume that *h*(*u*) is a real-valued bounded function and ${P_{\alpha _n} }h = {e^{ - {\alpha _n} }}\sum \nolimits _{k = 0}^\infty {h\left( k \right) \frac{{{{\alpha _n} ^k}}}{{k!}}}$. Consider the function *f*(.) which is a solution of the differential equation

$$\begin{aligned} {\alpha _n} f(x+1)-xf(x)=h(x)-P_{\alpha _n} h. \end{aligned}$$

Setting

$$\begin{aligned} h(x)=h_{A}(x)= {\left\{ \begin{array}{ll} 1, &\quad \text {if}\; x\in A,\\ 0, &\quad \text {if}\; x\notin A. \end{array}\right. } \end{aligned}$$

Putting $x=X$ and taking the expectation of both sides of the above differential equation, we have

$$\begin{aligned} F_{X}\left( A \right) - P_{\alpha _n} \left( A \right) = E\left[ {{\alpha _n} f\left( X+1 \right) - X f\left( X \right) } \right] . \end{aligned}$$

Thus, the problem of estimating $\Delta$ can be reduced to that of estimating the difference of the expectations

$$\begin{aligned} \left| {E{\alpha _n} f\left( X+1 \right) - E X f\left( X \right) } \right| . \end{aligned}$$

Before starting the main results in the next section we first recall the following remarkable lemmas:

### **Lemma 1**

(Barbour et al. 1992) *Let*$Vf_{A}\left( w \right) = f_{A}\left( w + 1 \right) - f_{A}\left( w \right) .$*Then, for*$A \subseteq \mathbb {Z_{+}}$*and*$k\in \mathbb {Z_{+}} \setminus \lbrace 0 \rbrace ,$

$$\begin{aligned} \mathop {sup}\limits _{w \ge k} \left| {V{f_A}\left( w \right) } \right| \le \min \left\{ {{{\alpha _n} ^{ - 1}}\left( {1 - {e^{ - {\alpha _n} }}} \right) ,\frac{1}{k}} \right\} . \end{aligned}$$

### **Lemma 2**

(Teerapabolarn and Wongkasem 2007) *Let*$w_{0} \in \mathbb {Z_{+}}$*and*$k\in \mathbb {Z_{+}} \setminus \lbrace 0 \rbrace ,$*we have*

$$\begin{aligned} \mathop {\sup }\limits _{w \ge k} \left| {V{f_{{C_{{w_0}}}}}\left( w \right) } \right| \le {{\gamma _n} ^{ - 1}}\left( {{e^{\gamma _n} } - 1} \right) \min \left\{ {\frac{1}{{{w_0} + 1}},\frac{1}{k}} \right\} . \end{aligned}$$

### **Lemma 3**

(Teerapabolarn 2009) *Let*$w_0 \in \mathbb {Z_{+}}$*and*$k\in \mathbb {Z_{+}} \setminus \lbrace 0, 1 \rbrace$. *Then, we have*

$$\begin{aligned} 0 < \mathop {\sup }\limits _{w \ge k} f\left( w \right) \le {{\bar{{\gamma _n}}} ^{ - 1}}\left( {{e^{\bar{{\gamma _n}}} } - 1} \right) \min \left\{ {\frac{1}{k},\frac{1}{{{w_0} + 1}}} \right\} . \end{aligned}$$

### **Lemma 4**

(Teerapabolarn 2013) *For*$w_0\in \mathbb {Z_{+}}$*and*$k\in \mathbb {Z_{+}}\setminus \lbrace 0, 1\rbrace$, *let*${p_{\bar{{\gamma _n}}} }\left( {{w_0}} \right) =\displaystyle \frac{{{e^{ - {\bar{{\gamma _n}}}}}{{\bar{{\gamma _n}}} ^{{w_0}}}}}{{{w_0}!}}$*and*${P_{\bar{{\gamma _n}}} }\left( {{w_0}} \right) =\displaystyle \sum \nolimits _{k = 0}^{{w_0}} {\displaystyle \frac{{{\bar{{\gamma _n}} ^k}{e^{ - {\bar{{\gamma _n}}} }}}}{{k!}}}$. *Then the following inequality is true*

$$\begin{aligned} \mathop {\sup }\limits _{w \ge k} {f_{{C_{{w_0}}}}}\left( w \right) \le \frac{{{P_{\bar{{\gamma _n}}} }\left( {{w_0}} \right) \left( {1 - {P_{\bar{{\gamma _n}}} }\left( {{w_0}} \right) } \right) }}{{{p_{\bar{{\gamma _n}}} }\left( {{w_0} + 1} \right) }}\min \left\{ {\frac{1}{{{w_0} + 1}},\frac{1}{k}} \right\} . \end{aligned}$$

## Results

Throughout the forthcoming, unless otherwise specified, we shall denote by $X_{n, 1},X_{n, 2}, \ldots ; n=1, 2, \ldots$ a row-wise triangular array of independent negative-binomial distributed random variables with probabilities

$$\begin{aligned} P\left( X_{n, i} = k \right) = C_{r_{n,i} + k - 1}^{k} \left( 1-p_{n, i} \right) ^{k}p_{n, i}^{r_{n,i}}, \end{aligned}$$

where $p_{n, i}\in (0,1); r_{n,i}=1, 2, \ldots ; i=1, 2, \ldots ; k=0, 1, \ldots .$ Let $W_{n}=\sum \nolimits _{i = 1}^{n} X_{n, i}$ and set ${\lambda _n} =E\left( W_{n} \right) =\sum \nolimits _{i = 1}^{n} r_{n,i} \left( 1-p_{n, i} \right) p_{n, i}^{-1}.$ Then, for ${r_{n,i}} \in \left\{ {1,2,\ldots .} \right\}$ we have the following theorems:

### **Theorem 1**

*For*$$A \subseteq \mathbb {Z_{+}},$$

$$\begin{aligned}&\mathop {\sup }\limits _A \left| {P(W_{n} \in A) - \sum \limits _{k \in A} {\frac{{{\lambda _n ^k}{e^{ - {\lambda _n} }}}}{{k!}}} } \right| \\&\quad \le \sum \limits _{i = 1}^n {\min \left\{ {\lambda _n^{ - 1}\left( {1 - {e^{ - {\lambda _n}}}} \right) {r_{n,i}}\left( {1 - {p_{n,i}}} \right) p_{n,i}^{ - 1},1 - p_{n,i}^{{r_{n,i}}}} \right\} \left( {1 - {p_{n,i}}} \right) p_{n,i}^{ - 1}}. \end{aligned}$$

### *Proof*

Let f and h are bounded real-valued functions defined on $\mathbb {Z_{+}}.$ For $w=0, 1,\ldots$ we have the Stein’s equation for Poisson distribution with a mean ${\lambda _n}$

$$\begin{aligned} {\lambda _n} f\left( w + 1 \right) - wf\left( w \right) = h\left( w \right) -P_{{\lambda _n} }\left( h \right) , \end{aligned}$$

where $$P_{{\lambda _n} }\left( h \right) = \displaystyle {e^{-{\lambda _n}}}\sum \nolimits _{k = 0}^{\infty } {h\left( k \right) } \frac{{{\lambda _n} ^{k}}}{k!}.$$

For $A \subseteq \mathbb {Z_{+}},$ let us denote by $h_{A}: \mathbb {Z_{+}} \rightarrow \mathbb {R}$ and by $f_{A}\left( w \right)$ the functions defined by

$$\begin{aligned} h_{A}(w)= {\left\{ \begin{array}{ll} 1, &{}\quad \text {if}\; w\in A,\\ 0, &{}\quad \text {if}\; w\notin A. \end{array}\right. } \end{aligned}$$

and

$$\begin{aligned} f_{A}(w)= {\left\{ \begin{array}{ll} \left( {w - 1} \right) !{{\lambda _n} ^{ - w}}{e^{\lambda _n} }\left[ {{P_{\lambda _n} }\left( {{h_{A \cap {C_{w - 1}}}}} \right) - {P_{\lambda _n} }\left( {{h_A}} \right) {P_{\lambda _n} }\left( {{h_{{C_{w - 1}}}}} \right) } \right] , &{} \quad \text {if}\; w\ge 1,\\ 0,&{} \quad \text {if}\; w=0, \end{array}\right. } \end{aligned}$$

where $$C_{w} = \left\{ 0,1,\cdots , w \right\} .$$

Given $f = {f_A}$ and $h={h_A}$, We have the following Stein’s equation:

$$\begin{aligned} {\lambda _n} f\left( {w + 1} \right) - wf\left( w \right) = {h_A}\left( w \right) - {P_{\lambda _n} }\left( {{h_A}} \right) , \end{aligned}$$

where

$$\begin{aligned} {P_{\lambda _n} }\left( {{h_A}} \right) = {e^{ - {\lambda _n} }}\sum \limits _{k = 0}^\infty {{h_A}\left( k \right) } \frac{{{\lambda _n ^k}}}{{k!}} = \sum \limits _{k \in A} {{e^{ - {\lambda _n} }}\frac{{{\lambda _n ^k}}}{{k!}}}. \end{aligned}$$

Therefore, the Stein’s equation can be written as follows:

$$\begin{aligned} {h_A}\left( w \right) - \sum \limits _{k \in A} {{e^{ - {\lambda _n} }}\frac{{{\lambda _n ^k}}}{{k!}}} = {\lambda _n} f\left( {w + 1} \right) - wf\left( w \right) . \end{aligned}$$

Taking expectations of both sides of above equation, we have

$$\begin{aligned} P(W_{n} \in A) - \sum \limits _{k \in A} {\frac{{{\lambda _n ^k}{e^{ - {\lambda _n} }}}}{{k!}}} =E[{\lambda _n} f(W_{n} + 1) - W_{n}f(W_{n})]. \end{aligned}$$

It follows that

8 $$\begin{aligned}&\mid P(W_{n}\in A)-\sum \limits _{k\in A}\frac{\lambda _{n}^{k}e^{-\lambda _{n}}}{k!}\mid\, = E[{\lambda _n} f(W_{n} + 1) - W_{n}f(W_{n})] \mid \nonumber \\&\quad \le \sum \limits _{i = 1}^n { \mid E[{r_{n,i}}({p^{-1}_{n,i}} - 1)f(W_n + 1) - {X_{n,i}}} f(W_{n})] \mid . \end{aligned}$$

Let $W_{i} = W_{n} - X_{n, i}.$ Then, for each *i*,  we get

$$\begin{aligned}&E[{r_{n,i}}({p^{-1}_{n,i}} - 1)f(W_n + 1) - {X_{n,i}}f(W_n)] \\&\quad =\displaystyle E[{r_{n,i}}({p^{-1}_{n,i}} - 1)f({W_i} + {X_{n,i}} + 1) - {X_{n,i}}f({W_i} + {X_{n,i}})] \\&\quad =\displaystyle E[E[({r_{n,i}}({p^{-1}_{n,i}} - 1)f({W_i} + {X_{n,i}} + 1) - {X_{n,i}}f({W_i} + {X_{n,i}}))|{X_{n,i}}]] \\&\quad =\displaystyle E[({r_{n,i}}({p^{-1}_{n,i}} - 1)f({W_i} + {X_{n,i}} + 1) - {X_{n,i}}f({W_i} + {X_{n,i}}))|{X_{n,i}} = 0]{p^{r_{n,i}}_{n,i}} \\&\qquad +\,\displaystyle E[({r_{n,i}}({p^{-1}_{n,i}} - 1)f({W_i} + {X_{n,i}} + 1) - {X_{n,i}}f({W_i} + {X_{n,i}}))|{X_{n,i}} = 1]{r_{n,i}}{p^{r_{n,i}}_{n,i}}(1 - {{p_{n,i}}}) \\&\qquad +\,\sum \limits _{k \ge 2} {E[\left( {r_{n,i}}({p^{-1}_{n,i}} - 1)f({W_i} + {X_{n,i}} + 1) \right. }\\&\quad {\left. -{X_{n,i}}f({W_i} + {X_{n,i}})\right) |{X_{n,i}} = k]} C_{{r_{n,i}} + k - 1}^k{p^{r_{n,i}}_{n,i}}{(1 - {{p_{n,i}}})^k} \\&\quad = E[{r_{n,i}}({p^{-1}_{n,i}} - 1){p^{r_{n,i}}_{n,i}}f({W_i} + 1)] \\&\qquad +\,E[{r_{n,i}^2}{\left( {1 - {{p_{n,i}}}} \right) ^2}{p^{{r_{n,i}} - 1}_{n,i}}f({W_i} + 2) - {r_{n,i}}{p^{r_{n,i}}_{n,i}}(1 - {{p_{n,i}}})f({W_i} + 1)] \\&\qquad +\,\sum \limits _{k \ge 2} {E[C_{{r_{n,i}} + k - 1}^k{r_{n,i}}(1 - {{p_{n,i}}}} {)^{k + 1}}{p^{{r_{n,i}} - 1}_{n,i}}f({W_i} + k + 1)\\&\qquad - kC_{{r_{n,i}} + k - 1}^k{p^{r_{n,i}}_{n,i}}{(1 - {{p_{n,i}}})^k}f({W_i} + k)] \\&\quad =\displaystyle {r_{n,i}}{\left( {1 - {{p_{n,i}}}} \right) ^2}{p^{{r_{n,i}} - 1}_{n,i}}E[f({W_i} + 1)] + E[{r_{n,i}^2}{(1 - {{p_{n,i}}})^2}{p^{{r_{n,i}} - 1}_{n,i}}f({W_i} + 2)] \\&\qquad +\,\sum \limits _{k \ge 2} {E[C_{{r_{n,i}} + k - 1}^k{r_{n,i}}(1 - {{p_{n,i}}}} {)^{k + 1}}{p^{{r_{n,i}} - 1}_{n,i}}f({W_i} + k + 1)\\&\qquad - kC_{{r_{n,i}} + k - 1}^k{p^{r_{n,i}}_{n,i}}{(1 - {{p_{n,i}}})^k}f({W_i} + k)] \\&\quad =\displaystyle {r_{n,i}}{\left( {1 - {{p_{n,i}}}} \right) ^2}{p^{{r_{n,i}} - 1}_{n,i}}E[f({W_i} + 1)] \\&\qquad +\,\sum \limits _{k \ge 2} {E[C_{{r_{n,i}} + k - 2}^{k - 1}{r_{n,i}}(1 - {{p_{n,i}}}} {)^k}{p^{{r_{n,i}} - 1}_{n,i}}f({W_i} + k) - kC_{{r_{n,i}} + k - 1}^k{p^{r_{n,i}}_{n,i}}{(1 - {{p_{n,i}}})^k}f({W_i} + k)] \\&\quad =\displaystyle {r_{n,i}}{\left( {1 - {{p_{n,i}}}} \right) ^2}{p^{{r_{n,i}} - 1}_{n,i}}E[f({W_i} + 1)] \\&\qquad +\,\sum \limits _{k \ge 2} {E[C_{{r_{n,i}} + k - 2}^{k - 1}{r_{n,i}}(1 - {{p_{n,i}}}} {)^k}{p^{{r_{n,i}} - 1}_{n,i}}f({W_i} + k)\\&\qquad -\,\left( {{r_{n,i}} + k - 1} \right) C_{{r_{n,i}} + k - 2}^{k - 1}{p^{r_{n,i}}_{n,i}}{(1 - {{p_{n,i}}})^k}f({W_i} + k)] \end{aligned}$$

$$\begin{aligned}&=\displaystyle {r_{n,i}}{\left( {1 - {p_{n,i}}} \right) ^2}p_{n,i}^{{r_{n,i}} - 1}E\left[ {f\left( {{W_i} + 1} \right) } \right] \\&\qquad +\,\displaystyle \sum \limits _{k \ge 2} {E\left[ {\frac{{{r_{n,i}} + k - 1}}{{r_{n,i}}}C_{{r_{n,i}} + k - 2}^{k - 1}{r_{n,i}}{{\left( {1 - {p_{n,i}}} \right) }^k}p_{n,i}^{{r_{n,i}} - 1}f\left( {{W_i} + k} \right) }\right. }\\&\qquad -\,{\left. { \left( {{r_{n,i}} + k - 1} \right) C_{{r_{n,i}} + k - 2}^{k - 1}p_i^{r_{n,i}}{{\left( {1 - {p_{n,i}}} \right) }^k}f\left( {{W_i} + k} \right) } \right] } \\&\qquad -\,\displaystyle \sum \limits _{k \ge 2} {\left( {\frac{{{r_{n,i}} + k - 1}}{{r_{n,i}}} - 1} \right) C_{{r_{n,i}} + k - 2}^{k - 1}{r_{n,i}}{{\left( {1 - {p_{n,i}}} \right) }^k}p_{n,i}^{{r_{n,i}} - 1}E\left[ {f\left( {{W_i} + k} \right) } \right] } \\&\quad = {r_{n,i}}{\left( {1 - {p_{n,i}}} \right) ^2}p_{n,i}^{{r_{n,i}} - 1}E[f({W_i} + 1)] \\&\qquad +\,\displaystyle \sum \limits _{k \ge 2} {\left( {{r_{n,i}} + k - 1} \right) C_{{r_{n,i}} + k - 2}^{k - 1}(1 - {p_{n,i}}} {)^{k + 1}}p_{n,i}^{{r_{n,i}} - 1}E\left[ {f({W_i} + k)} \right] \\&\qquad -\,\displaystyle \sum \limits _{k \ge 2} {\left( {\frac{{{r_{n,i}} + k}}{{r_{n,i}}} - 1} \right) C_{{r_{n,i}} + k - 1}^k{r_{n,i}}(1 - {p_{n,i}}} {)^{k + 1}}p_{n,i}^{{r_{n,i}} - 1}E\left[ {f({W_i} + k + 1)} \right] \\&\qquad -\,{r_{n,i}}{\left( {1 - {p_{n,i}}} \right) ^2}p_{n,i}^{{r_{n,i}} - 1}E[f({W_i} + 2)] \\&\quad =\displaystyle {r_{n,i}}{\left( {1 - {{p_{n,i}}}} \right) ^2}{p^{{r_{n,i}} - 1}_{n,i}}E[f({W_i} + 1)] - {r_{n,i}}{\left( {1 - {{p_{n,i}}}} \right) ^2}{p^{{r_{n,i}} - 1}_{n,i}}E[f({W_i} + 2)] \\&\qquad +\,\displaystyle \sum \limits _{k \ge 2} {kC_{{r_{n,i}} + k - 1}^k(1 - {{p_{n,i}}}} {)^{k + 1}}{p^{{r_{n,i}} - 1}_{n,i}}E\left[ {f({W_i} + k)} \right] \\&\qquad -\,\displaystyle \sum \limits _{k \ge 2} {kC_{{r_{n,i}} + k - 1}^k(1 - {{p_{n,i}}}} {)^{k + 1}}{p^{{r_{n,i}} - 1}_{n,i}}E\left[ {f({W_i} + k + 1)} \right] \\&\quad =\displaystyle {r_{n,i}}{\left( {1 - {{p_{n,i}}}} \right) ^2}{p^{{r_{n,i}} - 1}_{n,i}}E[f({W_i} + 1) - f({W_i} + 2)] \\&\qquad +\,\displaystyle \sum \limits _{k \ge 2} {kC_{{r_{n,i}} + k - 1}^k(1 - {{p_{n,i}}}} {)^{k + 1}}{p^{{r_{n,i}} - 1}_{n,i}}E[f({W_i} + k) - f({W_i} + k + 1)] \\&\quad =\displaystyle \sum \limits _{k \ge 1} {kC_{{r_{n,i}} + k - 1}^k(1 - {{p_{n,i}}}} {)^{k + 1}}{p^{{r_{n,i}} - 1}_{n,i}}E[f({W_i} + k) - f({W_i} + k + 1)] . \end{aligned}$$

By using Lemma 1, we have

9 $$\begin{aligned}&|E[{r_{n,i}}({p^{-1}_{n,i}} - 1)f(W_{n} + 1) - {X_{n,i}}f(W_{n})]|\nonumber \\&\quad \le \sum \limits _{k \ge 1} {kC_{{r_{n,i}} + k - 1}^k(1 - {{p_{n,i}}}} {)^{k + 1}}{p^{{r_{n,i}} - 1}_{n,i}}E|f({W_i} + k) - f({W_i} + k + 1)|\nonumber \\&\quad \le \sum \limits _{k \ge 1} {kC_{{r_{n,i}} + k - 1}^k(1 - {{p_{n,i}}}} {)^{k + 1}}{p^{{r_{n,i}} - 1}_{n,i}}\mathop {\sup }\limits _{w \ge k} |Vf(w)|\nonumber \\&\quad \le \min \left\{ {{{\lambda _n} ^{ - 1}}(1 - {e^{ - {\lambda _n} }}){p^{{r_{n,i}} - 1}_{n,i}}\sum \limits _{k \ge 1} {kC_{{r_{n,i}} + k - 1}^k(1 - {{p_{n,i}}}} {)^{k + 1}},} \right. \nonumber \\&\qquad \left. { {p^{{r_{n,i}} - 1}_{n,i}}\sum \limits _{k \ge 1} {C_{{r_{n,i}} + k - 1}^k(1 - {{p_{n,i}}}} {)^{k + 1}}} \right\} \nonumber \\&\quad =\min \left\{ {{{\lambda _n} ^{ - 1}}(1 - {e^{ - {\lambda _n} }}){p^{{r_{n,i}} - 1}_{n,i}}\left( {1 - {{p_{n,i}}}} \right) \sum \limits _{k \ge 1} {kC_{{r_{n,i}} + k - 1}^k(1 - {{p_{n,i}}}} {)^k},} \right. \nonumber \\&\qquad \left. {{p^{{r_{n,i}} - 1}_{n,i}}\left( {1 - {{p_{n,i}}}} \right) \left( {{p_{n,i}^{ - {r_{n,i}}}} - 1} \right) } \right\} \nonumber \\&\quad = \min \left\{ {\lambda _n^{ - 1}\left( {1 - {e^{ - {\lambda _n}}}} \right) p_{n,i}^{{r_{n,i}} - 1}\left( {1 - {p_{n,i}}} \right) {r_{n,i}}\left( {1 - {p_{n,i}}} \right) p_{n,i}^{ - {r_{n,i}} - 1}, p_{n.i}^{ - 1}\left( {1 - {p_{n,i}}} \right) \left( {1 -p_{n,i}^{r_{n,i}}} \right) } \right\} \nonumber \\&\quad = \min \left\{ {\lambda _n^{ - 1}\left( {1 - {e^{ - {\lambda _n}}}} \right) {r_{n,i}}\left( {1 - {p_{n,i}}} \right) p_{n,i}^{ - 1},1 - p_{n,i}^{{r_{n,i}}}} \right\} \left( {1 - {p_{n,i}}} \right) p_{n,i}^{ - 1}. \end{aligned}$$

To combine (8) and (9), we have

$$\begin{aligned}&\mathop {\sup }\limits _A \left| {P(W_{n} \in A) - \sum \limits _{k \in A} {\frac{{{\lambda _n ^k}{e^{ - {\lambda _n} }}}}{{k!}}} } \right| \\&\quad \le \sum \limits _{i = 1}^n {\min \left\{ {\lambda _n^{ - 1}\left( {1 - {e^{ - {\lambda _n}}}} \right) {r_{n,i}}\left( {1 - {p_{n,i}}} \right) p_{n,i}^{ - 1},1 - p_{n,i}^{{r_{n,i}}}} \right\} \left( {1 - {p_{n,i}}} \right) p_{n,i}^{ - 1}}. \end{aligned}$$

The proof is complete. $\square$

### *Remark 1*

It is easily seen that the (4) is a special case of the Theorem 1 with $r_{n,i} = 1; n=1,2,\ldots ; i = 1,2,\ldots n$

### **Theorem 2**

*Let*$W_{n}$*and*${\lambda _n}$*be defined as in Theorem* 1. *Then, for*$w_{0} \in \mathbb {N},$

$$\begin{aligned}&\left| {P(W_{n} \le w_0) - \sum \limits _{k \le w_0} {\frac{{{\lambda _n ^k}{e^{ - {\lambda _n} }}}}{{k!}}} } \right| \\&\quad \le {{\lambda _n} ^{ - 1}}\left( {{e^{\lambda _n} } - 1} \right) \sum \limits _{i = 1}^n {\min \left\{ {\frac{{{r_{n,i}}\left( {1 - {p_{n,i}}} \right) }}{{{p_{n,i}}\left( {{w_0} + 1} \right) }}, {1 - {p^{r_{n,i}}_{n,i}}}} \right\} \left( {1 - {p_{n,i}}} \right) p_{n,i}^{ - 1}}. \end{aligned}$$

### *Proof*

For $C_w =\lbrace 0,\ldots ,w\rbrace$ and $w_0 \in N$, let ${h_{w_0}}: \mathbb {Z_{+}} \rightarrow \mathbb {R} ,\,f_{C_{w_{0}}}(w_0)$ be defined by

$$\begin{aligned} {h_{C_{w_0}}}\left( w \right) = \left\{ \begin{array}{l} 1\quad\text{ if }\; w \le w_0, \\ 0\quad\text{ if }\;w > w_0. \\ \end{array} \right. \end{aligned}$$

$$\begin{aligned} {f_{{C_{{w_0}}}}}\left( w \right) = \left\{ { \begin{array}{ll} {\left( {w - 1} \right) !{{\lambda _n} ^{ - w}}{e^{\lambda _n} }\left[ {{P_{\lambda _n} }\left( {{h_{{C_{w0}}}}} \right) {P_{\lambda _n} }\left( {1 - {h_{{C_{w - 1}}}}} \right) } \right] } & {\text{ if }} \; {{w_0} < w,} \\ {\left( {w - 1} \right) !{{\lambda _n} ^{ - w}}{e^{\lambda _n} }\left[ {{P_{\lambda _n} }\left( {{h_{{C_{w - 1}}}}} \right) {P_{\lambda _n} }\left( {1 - {h_{{C_{{w_0}}}}}} \right) } \right] } & {\text{ if }} \; {{w_0} \ge w,} \\ 0 & {\text{ if }} \; {w = 0}. \\ \end{array}} \right. \end{aligned}$$

Given $f = f_{C_{w_0}}$ and $h=h_{C_{w_0}}.$ We have the Stein’s equation

$$\begin{aligned} {h_{C_{w_0}}}\left( w \right) - \sum \limits _{k \le w_0} {{e^{ - {\lambda _n} }}\frac{{{\lambda _n ^k}}}{{k!}}} = {\lambda _n} f\left( {w + 1} \right) - wf\left( w \right) . \end{aligned}$$

Taking expectations of both sides and arguing similarly to the proof of Theorem 1 we prove that

10 $$\begin{aligned} \left| {P(W_n \le w_0) - \sum \limits _{k \le w_0} {\frac{{{\lambda _n ^k}{e^{ - {\lambda _n} }}}}{{k!}}} } \right| \le \sum \limits _{i = 1}^n {|E[{r_{n,i}}({p^{-1}_{n,i}} - 1)f(W_n + 1) - {X_{n,i}}} f(W_{n})]|. \end{aligned}$$

According to the Theorem 1, we have

11 $$\begin{aligned}&E[{r_{n,i}}({p^{-1}_{n,i}} - 1)f(W_{n} + 1) - {X_{n,i}}f(W_{n})] \nonumber \\&\quad = \sum \limits _{k \ge 1} {kC_{{r_{n,i}} + k - 1}^k(1 - {{p_{n,i}}}} {)^{k + 1}}{p^{{r_{n,i}} - 1}_{n,i}}E[f({W_i} + k) - f({W_i} + k + 1)]. \end{aligned}$$

Hence, by (10), (11) and Lemma 2, we have

$$\begin{aligned}&\left| {P(W_{n} \le w_0) - \sum \limits _{k \le w_0} {\frac{{{\lambda _n ^k}{e^{ - {\lambda _n} }}}}{{k!}}} } \right| \le \sum \limits _{i = 1}^n {|E[{r_{n,i}}({p^{-1}_{n,i}} - 1)f(W_{n} + 1) - {X_{n,i}}} f(W_{n})]| \\&\quad \le \sum \limits _{i = 1}^n {\left( {\sum \limits _{k \ge 1} {kC_{{r_{n,i}} + k - 1}^k(1 - {{p_{n,i}}}} {)^{k + 1}}{p^{{r_{n,i}} - 1}_{n,i}}\mathop {\sup }\limits _{w \ge k} |Vf(w)|} \right) } \\&\quad \le \sum \limits _{i = 1}^n {\left( {{{\lambda _n} ^{ - 1}}\left( {{e^{\lambda _n} } - 1} \right) \min \left\{ {\dfrac{{{p^{{r_{n,i}} - 1}_{n,i}}}}{{{w_0} + 1}}\sum \limits _{k \ge 1} {kC_{{r_{n,i}} + k - 1}^k(1 - {{p_{n,i}}}} {)^{k + 1}},} \right. } \right. }\\&\qquad {\left. {\left. { {p^{{r_{n,i}} - 1}_{n,i}}\sum \limits _{k \ge 1} {C_{{r_{n,i}} + k - 1}^k(1 - {{p_{n,i}}}} {)^{k + 1}}} \right\} } \right) } \\&\quad \le \sum \limits _{i = 1}^n {\left( {{{\lambda _n} ^{ - 1}}\left( {{e^{\lambda _n} } - 1} \right) \min \left\{ {\dfrac{{{p^{{r_{n,i}} - 1}_{n,i}}\left( {1 - {{p_{n,i}}}} \right) }}{{{w_0} + 1}}\sum \limits _{k \ge 1} {kC_{{r_{n,i}} + k - 1}^k(1 - {{p_{n,i}}}} {)^k},} \right. } \right. }\\&\qquad {\left. {\left. {{p^{{r_{n,i}} - 1}_{n,i}}\left( {1 - {{p_{n,i}}}} \right) \left( {{p^{ - {r_{n,i}}}_{n,i}} - 1} \right) } \right\} } \right) } \\&\quad = \sum \limits _{i = 1}^n {\left( {\lambda _n^{ - 1}\left( {{e^{{\lambda _n}}} - 1} \right) \min \left\{ {\dfrac{{p_{n,i}^{{r_{n,i}} - 1}\left( {1 - {p_{n,i}}} \right) {r_{n,i}}\left( {1 - {p_{n,i}}} \right) }}{({w_0} + 1)p_{n,i}^{ {r_{n,i}} + 1}},\left( {1 - {p_{n,i}}} \right) p_{n,i}^{ - 1}\left( {1 - {p^{r_{n,i}}_{n,i}}} \right) } \right\} } \right) } \\&\quad = \sum \limits _{i = 1}^n {\left( {\lambda _n^{ - 1}\left( {{e^{{\lambda _n}}} - 1} \right) \min \left\{ {\dfrac{{{r_{n,i}}\left( {1 - {p_{n,i}}} \right) }}{{{p_{n,i}}\left( {{w_0} + 1} \right) }}, {1 - {p^{r_{n,i}}_{n,i}}}} \right\} \left( {1 - {p_{n,i}}} \right) p_{n,i}^{ - 1}} \right) }. \end{aligned}$$

Thus

$$\begin{aligned}&\left| {P(W_{n} \le w_0) - \sum \limits _{k \le w_0} {\frac{{{\lambda _n ^k}{e^{ - {\lambda _n} }}}}{{k!}}} } \right| \\&\quad \le {{\lambda _n} ^{ - 1}}\left( {{e^{\lambda _n} } - 1} \right) \sum \limits _{i = 1}^n {\min \left\{ {\frac{{{r_{n,i}}\left( {1 - {p_{n,i}}} \right) }}{{{p_{n,i}}\left( {{w_0} + 1} \right) }}, {1 - {p^{r_{n,i}}_{n,i}}}} \right\} \left( {1 - {p_{n,i}}} \right) p_{n,i}^{ - 1}}. \end{aligned}$$

This finishes the proof. $\square$

### *Remark 2*

It is easy to check that the (5) is a special case of Theorem 2 with $r_{n,i} = 1; n=1,2,\ldots ; i = 1,2,\ldots n$.

### **Theorem 3**

*Let*$W_{n} = \sum \nolimits _{i = 1}^{n} X_{i}$*and*${\bar{{\lambda _n}}} = \sum \nolimits _{i = 1}^{n} r_{n,i}q_{n,i}$*with*$q_{n,i}=1-p_{n,i}$. *Then, we have*

$$\begin{aligned} - {{\bar{{\lambda _n}}} ^{ - 1}}\left( {{e^{\bar{{\lambda _n}}} } - 1} \right) \sum \limits _{i = 1}^n {\min \left\{ {\alpha _i ,\frac{{\beta _i - \alpha _i }}{{{w_0} + 1}}} \right\} } \le P\left( {W_{n} \le {w_0}} \right) - \sum \limits _{k = 0}^{{w_0}} {\frac{{{\bar{{\lambda _n}} ^k}{e^{ - {\bar{{\lambda _n}}} }}}}{{k!}}} \le 0, \end{aligned}$$

*With*$$\alpha _i = 1 - {p^{r_{n,i}}_{n,i}} - {r_{n,i}}{{q_{n,i}}}{p^{r_{n,i}}_{n,i}}$$, $$\beta _i = {r_{n,i}}\left( {{p^{ - {r_{n,i}}}_{n,i}} - 1 - {r_{n,i}}{{q_{n,i}}}{p^{r_{n,i}}_{n,i}}} \right) .$$

### *Proof*

Arguing as in theorem (3), we have the Stein’s equation

$$\begin{aligned} {h_{w_0}}\left( w \right) - \sum \limits _{k =0}^{w_0} {{e^{ - {\bar{{\lambda _n}}} }}\frac{{{\bar{{\lambda _n}} ^k}}}{{k!}}} = {\bar{{\lambda _n}}} f\left( {w + 1} \right) - wf\left( w \right) . \end{aligned}$$

Taking expectations of both sides, we get

12 $$\begin{aligned}&\displaystyle P\left( {W_n \le {w_0}} \right) - \sum \limits _{k = 0}^{{w_0}} {\frac{{{\bar{{\lambda _n}} ^k}{e^{ - {\bar{{\lambda _n}}} }}}}{{k!}}}\nonumber \\&\quad = \displaystyle E\left[ {{\bar{{\lambda _n}}} f\left( {W_n + 1} \right) - W_n f\left( W_n \right) } \right] \nonumber \\&\quad = \sum \limits _{i = 1}^n {E\left[ {{{r_{n,i}}{q_{n,i}}}f\left( {W_n + 1} \right) - {X_{n,i}}f\left( W_n \right) } \right] }. \end{aligned}$$

Let ${W_i} = W_{n} - {X_{n,i}}$. Then, for each *i*, we deduce

$$\begin{aligned}&\displaystyle E[{r_{n,i}}{{q_{n,i}}}f(W_{n} + 1) - {X_{n,i}}f(W_{n})]\\&\quad =\displaystyle E[E[({r_{n,i}}{{q_{n,i}}}f({W_i} + {X_{n,i}} + 1) - {X_{n,i}}f({W_i} + {X_{n,i}}))|{X_{n,i}}]] \\&\quad = \displaystyle E\left[ {{r_{n,i}}{{q_{n,i}}}{p^{r_{n,i}}_{n,i}}f({W_i} + 1)} \right] + \displaystyle E[{r_{n,i}^2}{q^2_{n,i}}{p^{r_{n,i}}_{n,i}}f({W_i} + 2) - {r_{n,i}}{{q_{n,i}}}{p^{r_{n,i}}_{n,i}}f({W_i} + {X_{n,i}})]\\&\qquad + \displaystyle \sum \limits _{k \ge 2} {E[{r_{n,i}}C_{{r_{n,i}} + k - 1}^k{q^{k + 1}_{n,i}}{p^{r_{n,i}}_{n,i}}f({W_i} + k + 1) - kC_{{r_{n,i}} + k - 1}^k{q^k_{n,i}}{p^{r_{n,i}}_{n,i}}f({W_i} + k)]} \\&\quad = \displaystyle \sum \limits _{k \ge 2} {E[{r_{n,i}}C_{{r_{n,i}} + k - 2}^{k - 1}{q^k_{n,i}}{p^{r_{n,i}}_{n,i}}f({W_i} + k) - kC_{{r_{n,i}} + k - 1}^k{q^k_{n,i}}{p^{r_{n,i}}_{n,i}}f({W_i} + k)]} \\&\quad =\displaystyle \sum \limits _{k \ge 2} {E[\frac{{{r_{n,i}}k}}{{{r_{n,i}} + k - 1}}C_{{r_{n,i}} + k - 1}^k{q^k_{n,i}}{p^{r_{n,i}}_{n,i}}f({W_i} + k) - kC_{{r_{n,i}} + k - 1}^k{q^k_{n,i}}{p^{r_{n,i}}_{n,i}}f({W_i} + k)]} \\&\quad =\displaystyle \sum \limits _k {\frac{{k\left( {1 - k} \right) }}{{{r_{n,i}} + k - 1}}} C_{{r_{n,i}} + k - 1}^k{q^k_{n,i}}{p^{r_{n,i}}_{n,i}}f({W_i} + k) \\&\quad \ge \displaystyle - \sum \limits _k {\frac{{k\left( {k - 1} \right) }}{{{r_{n,i}} + k - 1}}} C_{{r_{n,i}} + k - 1}^k{q^k_{n,i}}{p^{r_{n,i}}_{n,i}}\mathop {\sup }\limits _{w \ge k} f\left( w \right) . \end{aligned}$$

By using Lemma 3, then we have

13 $$\begin{aligned}&-\displaystyle \sum \limits _k {\frac{{k\left( {k - 1} \right) }}{{{r_{n,i}} + k - 1}}} C_{{r_{n,i}} + k - 1}^k{q^k_{n,i}}{p^{r_{n,i}}_{n,i}}\mathop {\sup }\limits _{w \ge k} f\left( w \right) \nonumber \\&\quad \ge - {{\bar{{\lambda _n}}} ^{ - 1}}\left( {{e^{\bar{{\lambda _n}}} } - 1} \right) {p^{r_{n,i}}_{n,i}}\min \left\{ {\sum \limits _k {\frac{{k - 1}}{{{r_{n,i}} + k - 1}}} C_{{r_{n,i}} + k - 1}^k{q^k_{n,i}},} \right. \nonumber \\&\qquad \left. { \frac{1}{{{w_0} + 1}}\sum \limits _k {\frac{{k\left( {k - 1} \right) }}{{{r_{n,i}} + k - 1}}} C_{{r_{n,i}} + k - 1}^k{q^k_{n,i}}} \right\} . \end{aligned}$$

Moreover, we have

14 $$\begin{aligned} {p^{r_{n,i}}_{n,i}}\sum \limits _{k} {\frac{{k - 1}}{{{r_{n,i}} + k - 1}}} C_{{r_{n,i}} + k - 1}^k{q^k_{n,i}} \le 1 - {p^{r_{n,i}}_{n,i}} - {r_{n,i}}{{q_{n,i}}}{p^{r_{n,i}}_{n,i}} \end{aligned}$$

and

15 $$\begin{aligned}&\displaystyle {p^{r_{n,i}}_{n,i}}\sum \limits _{k} {\frac{{k\left( {k - 1} \right) }}{{{r_{n,i}} + k - 1}}} C_{{r_{n,i}} + k - 1}^k{q^k_{n,i}}\nonumber \\&\quad \le {r_{n,i}}\left( {{p^{ - {r_{n,i}}}_{n,i}} - 1 - {r_{n,i}}{{q_{n,i}}}{p^{r_{n,i}}_{n,i}}} \right) - \left( {1 - {p^{r_{n,i}}_{n,i}} - {r_{n,i}}{{q_{n,i}}}{p^{r_{n,i}}_{n,i}}} \right) . \end{aligned}$$

Hence, by (12), (13), (14) and (15), we can assert that

$$\begin{aligned} - {{\bar{{\lambda _n}}} ^{ - 1}}\left( {{e^{\bar{{\lambda _n}}} } - 1} \right) \sum \limits _{i = 1}^n {\min \left\{ {\alpha _i ,\frac{{\beta _i - \alpha _i }}{{{w_0} + 1}}} \right\} } \le P\left( {W_{n} \le {w_0}} \right) - \sum \limits _{k = 0}^{{w_0}} {\frac{{{\bar{{\lambda _n}} ^k}{e^{ - {\bar{{\lambda _n}}} }}}}{{k!}}} \le 0. \end{aligned}$$

The proof is complete. $\square$

### *Remark 3*

When ${r_{n,i}}=1$, we have

$$\begin{aligned} \begin{array}{l} \alpha _i = 1 - {{p_{n,i}}} - {{q_{n,i}}}{{p_{n,i}}} = \displaystyle \left( {1 - {{p_{n,i}}}} \right) \left( {1 - {{p_{n,i}}}} \right) = {q^2_{n,i}}, \\ \beta _i = {p^{-1}_{n,i}} - 1 - {{q_{n,i}}}{{p_{n,i}}} = \displaystyle \frac{{1 - {{p_{n,i}}}}}{{{{p_{n,i}}}}} - \left( {1 - {{p_{n,i}}}} \right) {{p_{n,i}}} = \displaystyle \frac{{\left( {1 - {{p_{n,i}}}} \right) \left( {1 - p_{n,i}^2} \right) }}{{{{p_{n,i}}}}} = {q^2_{n,i}}\frac{{1 + {{p_{n,i}}}}}{{{{p_{n,i}}}}}, \\ \beta _i - \alpha _i = \displaystyle {q^2_{n,i}}\left( {\frac{{1 + {{p_{n,i}}}}}{{{{p_{n,i}}}}} - 1} \right) = \frac{{{q^2_{n,i}}}}{{{{p_{n,i}}}}}. \\ \end{array} \end{aligned}$$

It is clear that the (6) is a special case of Theorem 3 with $r_{n,i} = 1; n=1,2,\ldots ; i = 1,2,\ldots n$.

### **Theorem 4**

*Let*$W_{n} = \sum \nolimits _{i = 1}^{n} X_{n,i}$*and*${\overline{{\lambda _n}}} = \sum \nolimits _{i = 1}^{n} r_{n,i} q_{i}$*with*${q_{n,i}}=1-{p_{n,i}}.$*Then, for*$w_0\in \mathbb {N}$*we have*

$$\begin{aligned} \left| {P\left( {W_{n} \le {w_0}} \right) - {P_{\overline{{\lambda _n}}} }\left( {{w_0}} \right) } \right| \le \frac{{{P_{\overline{{\lambda _n}}} }\left( {{w_0}} \right) \left( {1 - {P_{\overline{{\lambda _n}}} }\left( {{w_0}} \right) } \right) }}{{{p_{\overline{{\lambda _n}}} }\left( {{w_0} + 1} \right) }}\sum \limits _{i = 1}^n {\min \left\{ {{\alpha _i},\frac{{{\beta _i} - {\alpha _i}}}{{{w_0} + 1}}} \right\} , } \end{aligned}$$

*where*

$$\begin{aligned} \alpha _i = 1 - {p^{r_{n,i}}_{n,i}} - {r_{n,i}}{{q_{n,i}}}{p^{r_{n,i}}_{n,i}}\; { and }\; \beta _i = {r_{n,i}}\left( {{p^{ - {r_{n,i}}}_{n,i}} - 1 - {r_{n,i}}{{q_{n,i}}}{p^{r_{n,i}}_{n,i}}} \right) . \end{aligned}$$

### *Proof*

According to Theorem 3 we obtain the following inequality

$$\begin{aligned} \left| {P\left( {W_{n} \le {w_0}} \right) - {P_{\bar{{\lambda _n}}} }\left( {{w_0}} \right) } \right| \le \sum \limits _{i = 1}^n {\sum \limits _k {\frac{{k\left( {k - 1} \right) }}{{{r_{n,i}} + k - 1}}C_{{r_{n,i}} + k - 1}^k{q^k_{n,i}}{p^{r_{n,i}}_{n,i}}\mathop {\sup }\limits _{w \ge k} {f_{{C_{{w_0}}}}}\left( w \right) } }. \end{aligned}$$

By using Lemma 4, then we have

$$\begin{aligned}&\left| {P\left( {W_{n} \le {w_0}} \right) - {P_{\bar{{\lambda _n}}} }\left( {{w_0}} \right) } \right| \\&\quad \le \displaystyle \frac{{{P_{\bar{{\lambda _n}}} }\left( {{w_0}} \right) \left( {1 - {P_{\bar{{\lambda _n}}} }\left( {{w_0}} \right) } \right) }}{{{p_{\bar{{\lambda _n}}} }\left( {{w_0} + 1} \right) }}\sum \limits _{i = 1}^n {\sum \limits _k {\frac{{k\left( {k - 1} \right) }}{{{r_{n,i}} + k - 1}}C_{{r_{n,i}} + k - 1}^k{q^k_{n,i}}{p^{r_{n,i}}_{n,i}}} \min \left\{ {\frac{1}{{{w_0} + 1}},\frac{1}{k}} \right\} } \\&\quad \le \displaystyle \frac{{{P_{\bar{{\lambda _n}}} }\left( {{w_0}} \right) \left( {1 - {P_{\bar{{\lambda _n}}} }\left( {{w_0}} \right) } \right) }}{{{p_{\bar{{\lambda _n}}} }\left( {{w_0} + 1} \right) }}\sum \limits _{i = 1}^n {\min \left\{ {{\alpha _i},\frac{{{\beta _i} - {\alpha _i}}}{{{w_0} + 1}}} \right\} , } \\ \end{aligned}$$

with $$\alpha _i = 1 - {p^{r_{n,i}}_{n,i}} - {r_{n,i}}{{q_{n,i}}}{p^{r_{n,i}}_{n,i}}$$, $$\beta _i = {r_{n,i}}\left( {{p^{ - {r_{n,i}}}_{n,i}} - 1 - {r_{n,i}}{{q_{n,i}}}{p^{r_{n,i}}_{n,i}}} \right) .$$

Hence, the theorem is proved. $\square$

### *Remark 4*

In the same way as in Remarks 3, we notice that (7) is a special case of Theorem 4 with $r_{n,i} = 1; n=1,2,\ldots ; i = 1,2,\ldots n.$

## Conclusions

 We conclude this paper with the following comments. The received results in this paper are extensions and generalizations of results in Teerapabolarn and Wongkasem (2007), Teerapabolarn (2009, 2013). The results would be more interesting and valuable if the discussed negative-binomial random variables in this paper are dependent. We shall take this up in the next study.
